# Synthesis and characterization of the Anderson–Evans tungsto­anti­monate [Na_5_(H_2_O)_18_{(HOCH_2_)_2_CHNH_3_}_2_][SbW_6_O_24_]

**DOI:** 10.1107/S2053229621006239

**Published:** 2021-06-28

**Authors:** Kleanthi Sifaki, Nadiia I. Gumerova, Gerald Giester, Annette Rompel

**Affiliations:** a Universität Wien, Fakultät für Chemie, Institut für Biophysikalische Chemie, Althanstraße 14, 1090 Wien, Austria; b Universität Wien, Fakultät für Geowissenschaften, Geographie und Astronomie, Institut für Mineralogie und Kristallographie, Althanstraße 14, 1090 Wien, Austria

**Keywords:** polyoxometalate, POM, polyoxotungstate, POT, serinol, crystal structure, organic-inorganic hybrid

## Abstract

A novel tungsto­anti­monate, [Na_5_(H_2_O)_18_{(HOCH_2_)_2_CHNH_3_}_2_][Sb^V^W^VI^_6_O_24_] (**SbW_6_**), was synthesized from an aqueous solution and structurally characterized by single-crystal X-ray diffraction, which revealed *C*2/*c* symmetry. The structure contains two serinol [(HOCH_2_)_2_CHNH_3_]^+^ and five Na^+^ cations, which are octa­hedrally surrounded by 18 water mol­ecules, and one [Sb^V^W^VI^_6_O_24_]^7−^ anion. The serinol mol­ecules also play a critical role in the synthesis by acting as a mild buffering agent. Each of the W^VI^ and Sb^V^ ions is six-coordinated and displays a distorted octa­hedral motif. A three-dimensional supra­molecular framework is formed *via* hydrogen-bonding inter­actions between the tungsto­anti­monates and cations. Powder X-ray diffraction, elemental analysis, thermogravimetric analy­sis and IR spectroscopy were performed on **SbW_6_** to prove the purity, to identify the water content and to characterize the vibrational modes of the crystallized phase.

## Abstract 2

The isolation and characterization of the Anderson–Evans-type tungsto­anti­monate [Sb^V^W^VI^_6_O_24_]^7−^ with a T-shaped sodium–serinol com­plex, [Na_5_(H_2_O)_18_{(HOCH_2_)_2_CHNH_3_}_2_]^7+^, as counter-cation is reported.

## Introduction   

Polyoxometalates (POMs) are known as early transition metal–oxygen clusters (Pope, 1983 ▸; Gumerova & Rompel, 2020 ▸). They are assembled from {*M*O_*x*_} polyhedra, with *x* = 4–7 and *M* being commonly Mo^V/VI^, W^V/VI^, V^IV/V^, Nb^V^ or Ta^V^ as addenda ions, through sharing of corners and edges (Pope, 1983 ▸). POMs can be considered as either isopolyanions [*M_m_* O_*y*_]^*q*−^, which feature only one metallic *M* ion (Mo^V/VI^, W^V/VI^, V^IV/V^, Nb^V^ or Ta^V^), or heteropolyanions [*X_r_M_m_* O_*y*_]^*q*−^, which additionally contain a heteroelement *X* (Pope, 1983 ▸). POMs display a wide range of crucial applications, ranging from catalysis (Wang & Yang, 2015 ▸), materials science (Cherevan *et al.*, 2020 ▸) and mol­ecular magnetism (Clemente-Juan *et al.*, 2012 ▸), to bio- and nanotechnology (Rhule *et al.*, 1998 ▸; Bijelic *et al.*, 2018 ▸, 2019 ▸), as well as macromolecular crystallography (Bijelic & Rompel, 2015 ▸, 2017 ▸, 2018 ▸).

**Figure 1:**
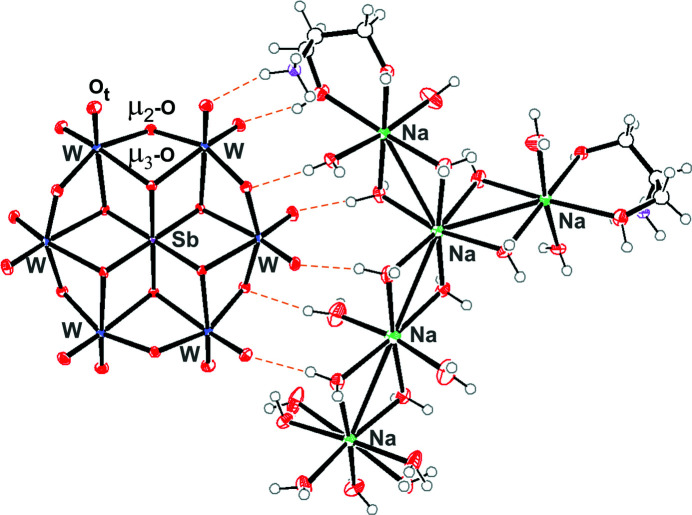
Displacement ellipsoid plot of **SbW_6_** with the hydrogen-bond inter­actions between the anion and the counter-cation com­plex highlighted (orange dashed line). Displacement ellipsoids are displayed at the 50% probability level. Colour code: W blue, Sb pink, Na green, C grey, N purple, O red and H white.

The Anderson–Evans polyoxoanion has the general for­mula [H_*y*_(*X*O_6_)*M*_6_O_18_]^*n−*^, where *y* = 0–6, *n* = 2–8, *M* = addenda ion (Mo^VI^ or W^VI^) and *X* = central heteroion in oxidation states from +2 to +7 (Blazevic & Rompel, 2016 ▸). Its structure consists of six corner- and edge-shared {MoO_6_} or {WO_6_} octa­hedra, which surround the {*X*O}_6_ octa­hedron (Evans, 1948 ▸). In the structure, there exist three differently coordinated oxygen ions (Fig. 1 ▸): six triple-bridged oxygen ions (μ_3_-O) that connect the heteroion and two addenda ions, six double-bridged oxygen ions (μ_2_-O) that connect two addenda ions and lastly two terminal oxygen ions (O_t_) per addenda ion (Evans, 1948 ▸; Pope, 1983 ▸). The oxidation state of a heteroion plays a significant role in the protonation mode of the triple-bridged oxygen ions (μ_3_-O) in the Anderson–Evans archetype, according to which they can be divided into three groups. The first, *i.e.* [*X^n^* ^+^ *M*_6_O_24_]^(12–*n*)−^ (*n* = 5–7), referred to as ‘type A’, is a deprotonated structure that exists when it contains hetero­ions with a high oxidation state (*e.g.* Te^VI^ or I^VII^). The second, *i.e.* [*X^n^* ^+^(OH)_6_ *M*_6_O_18_]^(6–*n*)−^ (*n* = 2–4), referred to as ‘type B’, is protonated on the six μ_3_-O ions, with each side having three protons. The B-type POMs are usually present when the heteroion has a low oxidation state (*e.g.* Ni^II^ or Co^II^) (Blazevic & Rompel, 2016 ▸). The third group, called ‘mixed type’, is a combination of the two types mentioned above, as it has protonated μ_3_-O ions only on one side (Gumerova *et al.*, 2019 ▸). Therefore, it is referred to as one-side protonated with one known polyoxotungstate example, [Cr^III^(OH)_3_W^VI^_6_O_21_]^6–^, so far (Gumerova *et al.*, 2019 ▸). Inter­estingly, there are some platinum-based com­pounds, with the general formula [H_*n*_Pt^IV^ *M* ^VI^_6_O_24_]^(8–*n*)−^ (*M* = W or Mo, where 1 < *n* < 6; Lee *et al.*, 2004 ▸), which exhibit protonation degrees of their μ_3_-O ions ranging from 1 to 6 and *n* is not necessarily an integer.

The majority of Anderson–Evans-type clusters have a planar hexa­gonal configuration with 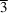 *m* (*D*_3*d*_) point symmetry, which is known as the α-isomer (Anderson, 1937 ▸). Another configuration of the Anderson–Evans-type cluster shows a bent structure of 2*mm* (*C*_2*v*_) point symmetry and is called the β-isomer (Lindqvist, 1959 ▸). An example of the β-isomer was presented by the Ogawa group (Ogawa *et al.*, 1988 ▸), *i.e.* [Sb^V^(OH)_2_Mo^VI^_6_O_22_]^5−^ and is one of the few reported structures to date (Lee & Sasaki, 1994 ▸; Zhang *et al.*, 2017 ▸; Li & Wei, 2021 ▸). The Anderson–Evans com­pounds can be modified in several ways: (i) by variation of the heteroion in the central position; (ii) by combination with various inorganic and organic cations, and (iii) by covalent attachment of one or two alkoxo ligands.

In recent decades, some unsubstituted tungsto­anti­monates, which have only one heteroion, Sb^III/V^, have been synthesized and structurally characterized (Table 1 ▸), but the majority of Sb-containing polyoxotungstates (POTs) contain additional heteroions, such as 3*d* or 4*f* metals (Tanuhadi *et al.*, 2018 ▸, 2020 ▸). Only three Sb-POTs of the Anderson–Evans archetype, namely, K_5_Na_2_[Sb^V^W^VI^_6_O_24_]·12H_2_O (Lee & Sasaki, 1987 ▸), K_5.5_H_1.5_[Sb^V^W^VI^_6_O_24_]·6H_2_O (Naruke & Yamase, 1992 ▸) and Na_7_[Sb^V^W^VI^_6_O_24_]·24H_2_O (Mukhacheva *et al.*, 2017 ▸), have been reported so far. Inter­estingly, the relative luminescence yield from the O→W LCMT transition of K_5.5_H_1.5_[Sb^V^W^VI^_6_O_24_]·6H_2_O was higher than that of Ln-containing Na_9_[Gd^III^(W^VI^_5_O_18_)_2_]·18H_2_O under the same conditions (Naruke & Yamase, 1992 ▸). The high luminescence yield of [Sb^V^W^VI^_6_O_24_]^7−^ is attractive for potential photochemical applications in the future.

Serinol (C_3_H_9_NO_2_, 2-amino­propane-1,3-diol) is a very stable, highly water soluble, nontoxic, odourless, biodegradable compound which is widely used as a versatile starting material in organic synthesis and as an additive for materials applications, such as com­posite materials (Barbera *et al.*, 2020 ▸; Andreessen & Steinbüchel, 2011 ▸). In POM synthesis, serinol can be seen as an alk­oxy­lation ligand or a counter-cation or buffering agent due to the presence of an amino group. Considering that the Sb-centred Anderson–Evans POT has not yet been reported with organic counter-cations, we expand the com­pound class by applying serinol, which can coordinate in different ways to metals through the –NH_2_ and –HOCH_2_ groups and thus significantly affects both the structure and properties, in the synthesis in Sb^5+^–WO_4_^2−^ (with an Sb:W ratio of 1:6) systems. Here we report a novel Anderson–Evans Sb-centred POT, [Na_5_(H_2_O)_18_{(HOCH_2_)_2_CHNH_3_}_2_][Sb^V^W^VI^_6_O_24_] (**SbW_6_**), being the first example of [Sb^V^W^VI^_6_O_24_]^7−^ crystallized with an organic counter-cation, which was synthesized from aqueous solution and has been fully characterized.

## Experimental   

### Synthesis and crystallization   

The reagents were used as purchased from Merck (Austria) and VWR (Austria) without further purification.

#### Synthesis of [Na_5_(H_2_O)_18_{(HOCH_2_)_2_CHNH_3_}_2_][SbW_6_O_24_] (SbW_6_)   

Na_2_WO_4_·2H_2_O (0.99 g, 3 mmol) and KSb^V^(OH)_6_ (0.13 g, 0.5 mmol) were mixed in a 6:1 ratio in H_2_O (12 ml), yielding a turbid solution. The solution was then acidified with aqueous HCl (1 *M*, 4.4 ml) and the pH was set at 4.0. Serinol [(HOCH_2_)_2_CHNH_2_; 0.18 g, 2 mmol] was then added and the pH was altered to 7.1. Under stirring and heating for 1 h, at 75 °C, the precipitate was dissolved, and the final solution was colourless. The pH after the reaction was 7.0. The solution was left for evaporation at room tem­perature, leading to colourless crystals suitable for single-crystal X-ray diffraction within 1 d (yield: 0.4 g, 60%, based on W). The pH of the Sb^5+^–WO_4_^2−^ solution was varied from 3.7 to 5.0; however, after the addition of serinol (0.18 g, 2 mmol), the final pH was in the range from 7.0 to 7.7 and, in all cases, crystals with the same unit cell were obtained. Other synthetic routes, such as reflux reaction and hydro­thermal synthesis at 120 °C, for the same reaction mixture led to the same product. Elemental analysis found (calculated) for [Na_5_(H_2_O)_18_{(HOCH_2_)_2_CHNH_3_}_2_][Sb^V^W^VI^_6_O_24_] (%): C 3.18 (3.23), H 2.56 (2.53), N 1.29 (1.25), O 32.89 (32.97). FT–IR (cm^−1^): 3357 (*s*), 2952 (*sh*), 1610 (*s*), 1498 (*s*), 1464 (*sh*), 1373 (*w*), 1256 (*w*), 1099 (*w*), 1037 (*s*), 1018 (*sh*), 927 (*s*), 850 (*s*), 703 (*sh*), 632 (*w*), 640 (*w*), 563 (*w*), 420 (*s*), 349 (*s*), 310 (*s*).

### IR spectroscopy   

**SbW_6_** was characterized by IR spectroscopy on a Bruker Vertex70 IR Spectrometer equipped with a single-reflection diamond-ATR unit in the range 4000–300 cm^−1^.

### TGA measurements   

Thermogravimetric analysis (TGA) was performed on a Mettler SDTA851e Thermogravimetric Analyzer under a nitro­gen flow with a heating rate of 5 K min^−1^ in the region from 303 to 873 K.

### Elemental analysis   

The determination of C/H/N/O was carried out using an ‘EA 1108 CHNS-O’ elemental analyzer by Carlo Erba Instruments at the Mikroanalytisches Laboratorium, Faculty of Chemistry, University of Vienna.

### Powder X-ray diffraction (PXRD)   

PXRD was performed on a Bruker D8 Advance diffrac­tometer, with Cu *K*α radiation (λ = 1.54056 Å), a Lynxeye silicon strip detector and a SolX energy dispersive detector (variable slit aperture with 12 mm, 10° ≤ 2θ ≤ 50°).

### Refinement   

In Table 2 ▸, the crystallographic characteristics of **SbW_6_** and the experimental conditions of data collection and refinement are reported. The positions of the H atoms of the water mol­ecules were obtained by difference Fourier techniques and were refined with free isotropic displacement parameters and O—H distances restrained to 0.95 (2) Å. The disordered water mol­ecule in the coordination sphere of atom Na1 was refined with two positions (O23 and O24), with free occupancy factors to a total of 100%. The H atoms of this disordered group had *U*_iso_(H) values set to 1.5*U*_eq_(O) of the parent atom. H atoms bound to N or C atoms were placed in idealized positions (N—H = 0.91 Å and C—H = 0.99 or 1.00 Å for CH_2_ and CH groups, respectively) and refined in riding modes, with *U*_iso_(H) values set to 1.5*U*_eq_(N) or to 1.2*U*_eq_(C).

## Results and discussion   

The preparation of **SbW_6_** was carried out at a W^VI^ to Sb^V^ ratio of 6:1 and at a pH of 7.1. In the absence of serinol, at pH 7.5, protonated K_5.5_H_1.5_[Sb^V^W^VI^_6_O_24_]·6H_2_O (Naruke & Yamase, 1992 ▸), and at pH 4.5, unprotonated K_5_Na_2_[Sb^V^W^VI^_6_O_24_]·12H_2_O (Lee & Sasaki, 1987 ▸), were obtained.

The main structural elements of **SbW_6_** are the Anderson–Evans [Sb^V^W^VI^_6_O_24_]^7−^ anion and the com­plex [Na_5_(H_2_O)_18_{(HOCH_2_)_2_CHNH_3_}_2_]^7+^ cation, which are connected *via* hydrogen bonds between terminal (O_t_) and bridging O atoms (μ_2_-O) of the polyanion and protons from the cationic com­plex (Fig. 1 ▸). Crystallographically centrosymmetric [Sb^V^W^VI^_6_O_24_]^7−^ shows the characteristic Anderson–Evans A-type structure with a central {SbO_6_} octa­hedron surrounded by six edge-shared {WO_6_} octa­hedra that form a planar array of distorted octa­hedra (Fig. 1 ▸). The average W—Sb bond length is 3.26 Å. As is typical for all Anderson–Evans A-type structures, three different coordination modes of the O atoms are present in the structure: six triple-bridged oxygen ions (μ_3_-O) connect the heteroion and two W ions, six double-bridged oxygen ions (μ_2_-O) connect two W ions and two terminal oxygen (O_t_) ions are connected to each of the six W ions (Fig. 1 ▸). The average distance for Sb—μ_3_-O is 1.98 Å, for W—μ_3_-O is 2.27 Å, for W—μ_2_-O is 1.94 Å and for W—O_t_ is 1.74 Å. The values are com­parable with those of K_5.5_H_1.5_[Sb^V^W^VI^_6_O_24_] (Naruke & Yamase, 1992 ▸). For instance, the Sb—μ_3_-O bond length (1.98 Å) differs by only 0.03 Å from the others reported by Naruke & Yamase (1992 ▸) (2.01 Å). Applying bond valence sum (BVS) calculations (Brown & Altermatt, 1985 ▸), all the W ions in [Sb^V^W^VI^_6_O_24_]^7−^ exhibit the +VI oxidation state (average calculated value of 6.01) and Sb shows the +V oxidation state (5.37). Based on BVS analysis and the number of counter-cations, it was concluded that the Anderson–Evans anion is not protonated and belongs to type A.

The counter-cation is com­posed of five octa­hedrally coordinated Na^+^ ions, which assemble in an elevated T-shape form, and two protonated serinol mol­ecules that are coordinated to two Na^+^ ions *via* –HOCH_2_ groups. This group has crystallographically imposed twofold symmetry. One serinol ligand inter­acts through the –NH_3_ group with the terminal O atom of the POT anion, and the second inter­acts with the adjacent O atom in {NaO_6_} (Fig. 1 ▸).

**Figure 2:**
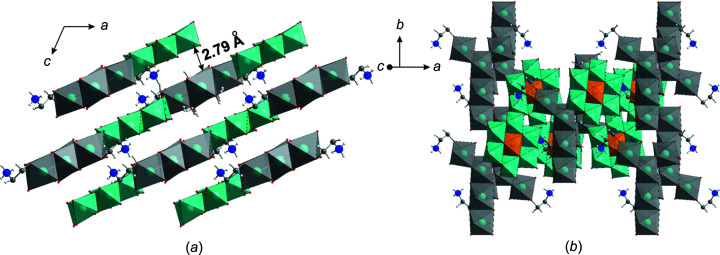
The crystal packing of **SbW_6_**, (*a*) viewed along the *b* axis and (*b*) viewed along the *z* axis. Colour code: {WO_6_} turquoise, {SbO_6_} orange, {Na(H_2_O)_6_} grey, C grey, N blue, O red and H black.

The three-dimensional (3D) structure of **SbW_6_** consists of two-dimensional (2D) sheets formed of [Sb^V^W^VI^_6_O_24_]^7−^ anions and com­plex [Na_5_(H_2_O)_18_{(HOCH_2_)_2_CHNH_3_}_2_]^7+^, cations connected *via* hydrogen bonds (Figs. 1 ▸ and 2 ▸). The distances between 2D layers are approximately 2.79 Å, which allows the formation of hydrogen bonds between the layers and creates cavities along the *b* axis (Fig. 2 ▸ *a*). This packing is different to that observed in K_5.5_H_1.5_[Sb^V^W^VI^_6_O_24_]·6H_2_O and K_5_Na_2_[Sb^V^W^VI^_6_O_24_]·12H_2_O, where the layers of anions alternate with layers or single polyhedra of counter-cations.

**Figure 3:**
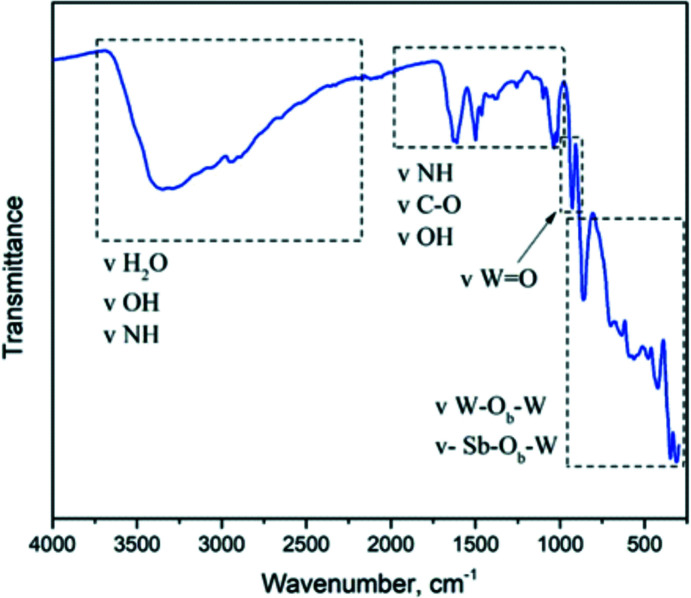
IR spectrum of **SbW_6_** in the region from 4000 to 300 cm^−1^.

The IR spectrum of **SbW_6_** (Fig. 3 ▸) is characteristic for Anderson–Evans POMs (Liu *et al.*, 2015 ▸; Qu *et al.*, 2012 ▸). The broad bands in the region between 2300 to 3750 cm^−1^ represent the vibrations of the –OH groups of H_2_O and the N—H bonds of the amine groups of serinol. In the area between 1610 and 1018 cm^−1^, the bands are attributed to the vibrations of C—H, C—O and again N—H in serinol. The bands at about 930 and 880 cm^−1^ are attributed to anti­symmetric stretching vibrations of the terminal W=O bonds and Sb—O—W bridges (O_b_), respectively. The bands at 640 and 563 cm^−1^ are associated with the asymmetric stretching of W—O—W bridges (O_b_) and the bending vibrations of W—O—W, respectively. Lastly, the bands between 750 and 300 cm^−1^ are contributed by Sb—O—W vibrations (Liu *et al.*, 2015 ▸).

**Figure 4:**
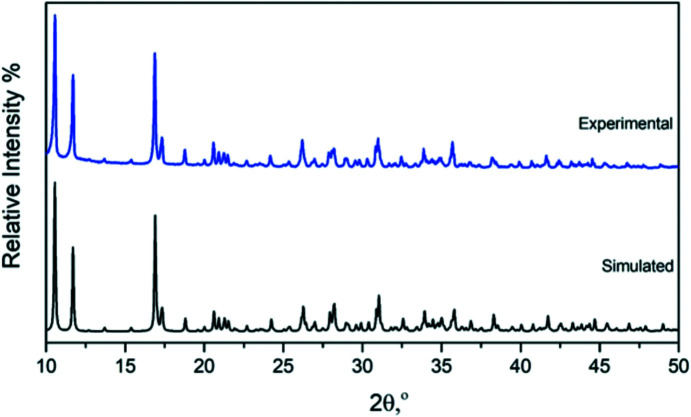
Experimental (blue colour) and simulated (black colour) powder XRD pattern of **SbW_6_**.

The powder XRD pattern of **SbW_6_** (Fig. 4 ▸) was investigated at room tem­perature. The simulated powder diffraction pattern was based on the single-crystal structural data. The observed peak positions are in good alignment with the simulated patterns, which confirms that the POT structure had been solved accurately and that **SbW_6_** consists of a single phase.

**Figure 5:**
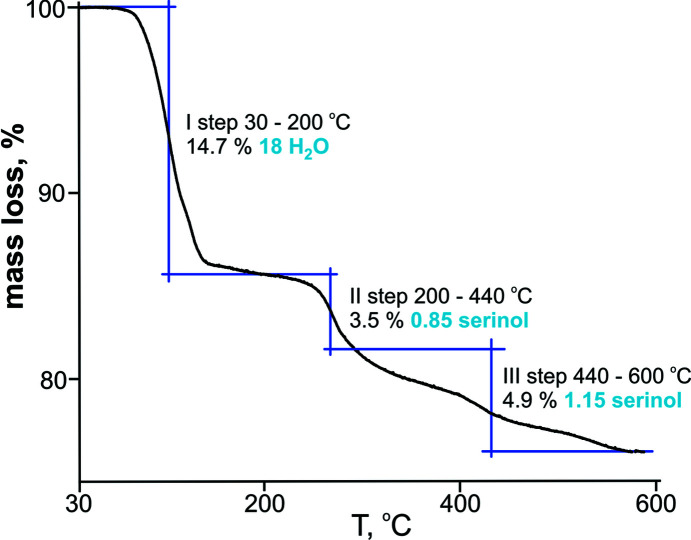
Thermogravimetric curve of **SbW_6_**.

The exact number of water mol­ecules was determined using TGA. The curve (Fig. 5 ▸) shows three weight-loss steps during the heating process from 30 to 600 °C. The first weight loss of 14.7% in the tem­perature range 30–200 °C corresponds to all water mol­ecules from the Na^+^ coordinating spheres. The second and third step correspond in total to 8.4% and the loss of two serinol mol­ecules.

The com­position of the counter-cation has remarkable effects on the crystal packing and thus on the physical properties of Anderson–Evans POMs (Blazevic & Rompel, 2016 ▸). The success in synthesizing **SbW_6_** shows that the Sb-centred Anderson–Evans POT is a versatile building block, which can be modified by organic counter-cations into high-dimensional architectures. **SbW_6_** is the first reported K^+^-free salt with an organic counter-cation, and it has much higher water solubility and can expand the areas of its application in aqueous solution.

## Supplementary Material

Crystal structure: contains datablock(s) I, global. DOI: 10.1107/S2053229621006239/ky3204sup1.cif

Structure factors: contains datablock(s) I. DOI: 10.1107/S2053229621006239/ky3204Isup2.hkl

Chemical scheme for the title compound. DOI: 10.1107/S2053229621006239/ky3204sup3.pdf

CCDC reference: 2079597

## Figures and Tables

**Table 1:** POTs with Sb^III/V^ as the only heteroion [based on the Inorganic Crystal Structure Database (FIZ, Karlsruhe; http://www.fiz-informationsdienste.de/DB/icsd/www-recherche.htm) and the Cambridge Structural Database (CSD; Groom *et al.*, 2016 ▸) in April 2021]

POT	Type	References
K_5_Na_2_[Sb^V^W^VI^_6_O_24_]	Anderson	Lee & Sasaki (1987 ▸)
K_5.5_H_1.5_[Sb^V^W^VI^_6_O_24_]	Anderson	Naruke & Yamase (1992 ▸)
Na_7_[Sb^V^W^VI^_6_O_24_]	Anderson	Mukhacheva *et al.* (2017 ▸)
K_6_[H_12_Sb^V^_6_W^VI^_4_O_36_]	Pseudo-Anderson–Evans dimer	Park *et al.* (1994 ▸)
(NH_4_)_9_[Sb^V^W^VI^_18_O_60_(OH)_2_]	Dawson	Zhang *et al.* (2010 ▸)
Na_9_[Sb^III^W^VI^_9_O_33_]	Keggin	Bösing *et al.* (1997 ▸)
K_12_[Sb^III^_2_W^VI^_22_O_74_(OH)_2_]	Krebs	Bösing *et al.* (1997 ▸)
[N(CH_3_)_4_]_10_Na_12_[Na_2_Sb^III^_8_W^VI^_36_O_132_(H_2_O)_4_]	Trimer based on lacunary Keggin	Bösing *et al.* (1997 ▸)
(H_2_en)_8_H_6_{[Sb^III^_2_(W^VI^O_2_)_2_(*B*-β-Sb^III^W^VI^_9_O_33_)_2_][(W^VI^O_2_)_2_(W^VI^O_3_)_2_(*B*-β-Sb^III^W^VI^_9_O_33_)_2_]} (en = ethyl­enedi­amine)	Krebs	Xin *et al.* (2019 ▸)
K_11_Na_16_[H_2_(Sb^III^W^VI^_9_O_33_)(W^VI^_5_O_12_)(Sb^III^_2_W^VI^_29_O_103_)]	Trimer based on lacunary Keggin	Tanuhadi *et al.* (2021 ▸)

**Table 2:** Experimental details

Crystal data	
Chemical formula	[Na_5_(H_2_O)_18_(C_3_H_10_NO_2_)_2_][SbW_6_O_24_]
*M*_r_	2232.32
Crystal system, space group	Monoclinic, *C*2/*c*
Temperature (K)	200
*a*, *b*, *c* (Å)	21.9761 (14), 13.9179 (9), 16.209 (1)
β (°)	111.189 (2)
*V* (Å^3^)	4622.5 (5)
*Z*	4
Radiation type	Mo *K*α
μ (mm^−1^)	15.61
Crystal size (mm)	0.1 × 0.08 × 0.05

Data collection	
Diffractometer	Bruker APEXII CCD
Absorption correction	Multi-scan (*SADABS*; Bruker, 2016 ▸)
*T*_min_, *T*_max_	0.542, 0.747
No. of measured, independent and observed [*I* > 2σ(*I*)] reflections	100214, 8841, 8213
*R*_int_	0.030
(sin θ/λ)_max_ (Å^−1^)	0.770

Refinement	
*R*[*F* ^2^ > 2σ(*F* ^2^)], *wR*(*F* ^2^), *S*	0.013, 0.025, 1.15
No. of reflections	8841
No. of parameters	390
No. of restraints	23
H-atom treatment	H atoms treated by a mixture of independent and constrained refinement
Δρ_max_, Δρ_min_ (e Å^−3^)	0.60, −0.69

Computer programs: *APEX3* (Bruker, 2015 ▸), *SAINT* (Bruker, 2016 ▸), *SHELXT2018* (Sheldrick, 2015*a* ▸), *shelXle* (Hübschle *et al.*, 2011 ▸), *SHELXL2018* (Sheldrick, 2015*b* ▸), *OLEX2* (Dolomanov *et al.*, 2009 ▸), *DIAMOND* (Brandenburg, 2006 ▸), *OLEX2* (Dolomanov *et al.*, 2009 ▸) and *PLATON* (Spek, 2020 ▸).
