# Supplementary material for: Synthesis and characterization of the Anderson–Evans tungsto­anti­monate [Na5(H2O)18{(HOCH2)2CHNH3}2][SbW6O24]
Source: Acta Crystallogr C Struct Chem. 2021 Jun 28;77(Pt 7):420–5. doi: 10.1107/S2053229621006239 (PMC8254527; doi:10.1107/S2053229621006239)

STRUCTURAL CHEMISTRY

Volume 77 (2021)

Supporting information for article:

Synthesis and characterization of the Anderson–Evans tungstoantimonate $[Na_5(H_2O)_{18}\{(HOCH_2)_2CHNH_3\}_2][SbW_6O_{24}]$

Kleanthi Sifaki, Nadiia I. Gumerova, Gerald Giester and Annette Rompel

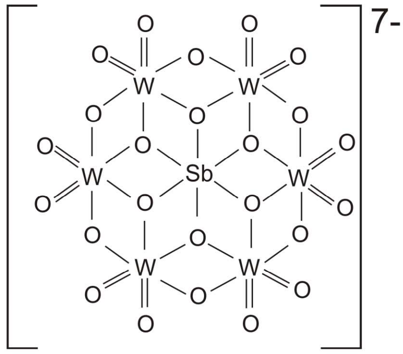

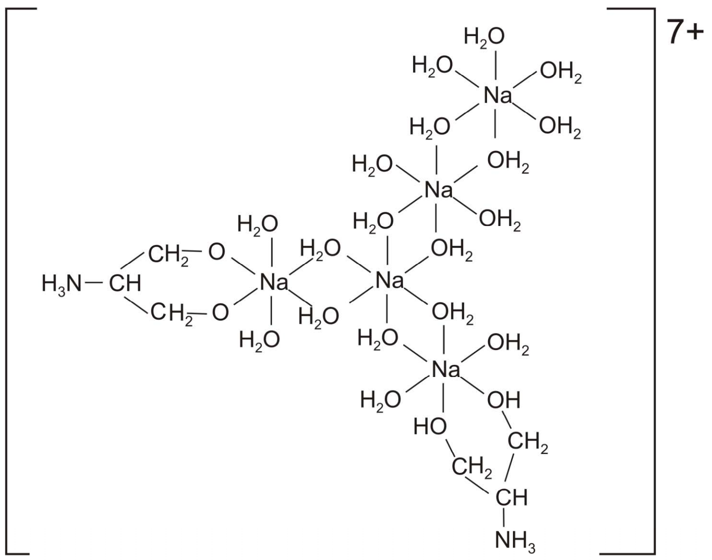

Supplement: Supplementary file 3 [file c-77-00420-sup3.pdf]
